# Refractory Anti‐HMGCR Necrotizing Myopathy With Progressive Hypercapnic Respiratory Failure Responsive to a JAK Inhibitor

**DOI:** 10.1155/crrh/7268721

**Published:** 2026-10-18

**Authors:** N. Huang, T. B. George, L. Christopher-Stine, A. Nicolas, C. Oddis, D. P. Ascherman, V. M. Liarski

**Affiliations:** ^1^ University of Pittsburgh Myositis Center Division of Rheumatology and Clinical Immunology University of Pittsburgh School of Medicine Pittsburgh Pennsylvania USA pitt.edu; ^2^ Johns Hopkins University School of Medicine Division of Rheumatology Johns Hopkins University School of Medicine Baltimore Maryland USA jhu.edu

**Keywords:** 3-hydroxy-3-methylglutaryl-coenzyme a reductase (HMGCR) antibody, case report, immune-mediated necrotizing myopathy, tofacitinib

## Abstract

Immune‐mediated necrotizing myopathy (IMNM) associated with 3‐hydroxy‐3‐methylglutaryl‐coenzyme A reductase (HMGCR) antibodies is a rare condition often associated with statin use. It typically presents with proximal weakness and markedly elevated creatine phosphokinase (CPK) levels and is often refractory, requiring long‐term immunotherapy. Respiratory involvement is uncommon but can be life‐threatening. Herein, we report the case of a 73‐year‐old woman with long‐standing anti‐HMGCR antibody–positive IMNM, refractory to glucocorticoids, methotrexate, azathioprine, intravenous immunoglobulin (IVIg), rituximab, and plasma exchange (PLEX), who presented with progressive hypercapnic respiratory failure. Despite ongoing treatment with IVIg, PLEX, and high‐dose glucocorticoids, the patient’s respiratory function worsened, and she required mechanical ventilation (MV). The addition of tofacitinib, a Janus kinase (JAK) inhibitor, coincided with marked clinical improvement, discontinuation of MV, and successful transition to room air. The patient continued to do well on this therapy, with improvement in muscle strength until outpatient discharge. The clinical benefit of JAK inhibition has been reported in dermatomyositis, overlap myositis, and anti–MDA5‐positive disease, particularly in refractory cases with pulmonary or systemic involvement—but has not been described in the setting of refractory IMNM. In our patient, who had exhausted multiple conventional therapies and PLEX, the rapid clinical improvement after JAK inhibition strongly suggests a therapeutic effect and highlights the need for future studies of JAK inhibitors in IMNM.

## 1. Introduction

Immune‐mediated necrotizing myopathy (IMNM) represents a subgroup of idiopathic inflammatory myopathies (IIMs) characterized by proximal muscle weakness, muscle necrosis with minimal lymphocytic inflammation on muscle biopsy [1, 2], and autoantibody positivity targeting 3‐hydroxy‐3‐methylglutaryl‐coenzyme A reductase (HMGCR) [3, 4]. Often related to statin exposure, anti‐HMGCR + IMNM is frequently refractory to treatment and prone to relapse—particularly in younger patients who may have more severe disease. Available therapies typically include intravenous immunoglobulin (IVIg), often in combination with glucocorticoids and other immunosuppressive agents such as methotrexate, azathioprine, or rituximab. Additional interventions may include plasma exchange (PLEX) [5–7]. In IMNM, muscle damage with fatty replacement leads to chronic muscle weakness unresponsive to additional immunosuppressive or immunomodulatory therapy, which presents an ongoing management challenge to rheumatologists. Although JAK inhibition has been reported in other inflammatory myopathies including dermatomyositis, overlap myositis, and anti‐MDA5–positive disease [8], it has not been described in the setting of refractory IMNM. Additionally, this case underscores the importance of assessing global disease activity, not limited to serum CPK levels, in advanced IMNM.

## 2. Case Presentation

### 2.1. IMNM Presentation, Diagnosis, and Prior Treatment

A 58‐year‐old woman with a history of gout, osteoporosis, diverticulitis, mixed hyperlipidemia, and hypertension was diagnosed in 2010 with anti‐HMGCR–positive IMNM. Her initial exposure was to atorvastatin in 2004. She reported upper extremity weakness, and although atorvastatin was stopped in 2005, she continued to experience weakness with only modest subjective improvement (∼50%), and persistent muscle pain. In 2006, ezetimibe was begun but was stopped after 1 month because of recurrent symptoms. Pravastatin was then started in 2007, but myalgias and worsening muscle weakness associated with a CPK level of 1897 U/L led to discontinuation after 3 months (Figure 1).

**FIGURE 1:**
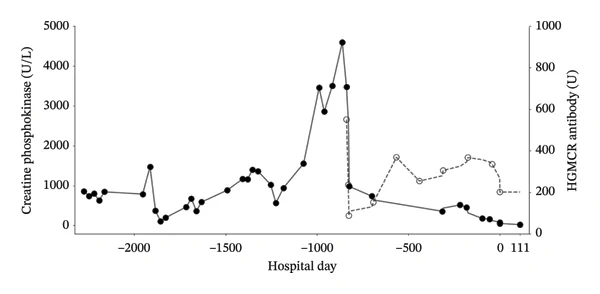
Levels of creatinine phosphokinase (CPK) and 3‐hydroxy‐3‐methylglutaryl‐coenzyme A reductase (HMGCR) antibody since the diagnosis of immune‐mediated necrotizing myopathy (IMNM). CPK data points are denoted with black circles and a black solid line and HMGCR data points as open circles and a dashed line. Hospital day 0 is defined as the date of presentation to the emergency room, leading to index patient hospitalization.

Serological studies included a negative antinuclear antibody (ANA), SSA, SSB, Smith, and Scl‐70 autoantibodies. Electromyography (EMG) performed in 2008 was reported as normal. Shortly thereafter, at the time of a cholecystectomy, the CPK ranged from 600 to 3000 U/L, subsequently rising to 4462 U/L and later 8728 U/L, both in 2009. A repeat EMG in November 2008 was normal, and a concurrent needle muscle biopsy revealed myopathic and neurogenic changes without inflammatory infiltrates.

The patient was then evaluated at an academic myositis referral center, where magnetic resonance imaging (MRI) showed marked muscle edema in all three lower extremity muscle compartments with minimal atrophy, raising suspicion for an immune‐mediated myopathy. A surgical muscle biopsy of the left rectus femoris in 2010 revealed extensive myofiber degeneration, necrosis, and myophagocytosis consistent with a necrotizing myopathy. The patient was later found to be anti‐HMGCR antibody positive. There was no family history of acquired or inherited muscle disease.

A combination of glucocorticoids and azathioprine was ineffective, prompting the addition of monthly IVIg (2 g/kg Gammagard) in 2010 that resulted in normalization of CPK levels (for the first time). Azathioprine was subsequently discontinued, and IVIg was tapered to every 3 months. However, after a rise in the CPK in 2012, monthly infusions of Gamunex were begun in place of Gammagard.

Despite these interventions, the CPK level continued to rise, peaking at 11,166 U/L in 2013 with worsening muscle weakness. Rituximab (two 1‐g doses 2 weeks apart) was therefore administered in 2013 along with monthly Gammagard. A repeat MRI demonstrated improvement with reduced muscle edema in the lower extremities without significant atrophy or fibrosis. The CPK never normalized (∼5000 U/L), and rituximab was deemed minimally effective. The frequency of Gammagard was then increased to every 2 weeks (2 g/kg), with eventual stabilization of muscle strength and reduction in CPK to the range of 1000–1500 U/L. The patient’s neuromuscular examination revealed 4‐/5 neck flexor and hip flexor weakness with otherwise normal proximal and distal muscle testing. Her disease remained stable until 2022, when the CPK rose in conjunction with worsening hip flexor weakness, leading to the initiation of methotrexate, which was poorly tolerated. A rise in the CPK (3471 U/L), worsening weakness, and a level of anti‐HMGCR antibody beyond the detectable range (> 550 U) prompted hospitalization and PLEX in 2023 followed by another 1‐g dose of rituximab. She developed severe *Clostridium difficile* infection and a T12 osteoporotic compression fracture, precluding further rituximab dosing. However, Gammagard was continued. The CPK improved to 735 U/L 6 months after her hospitalization and decreased further to 161 U/L later in 2024, but HMGCR antibody titers increased from 87 U after PLEX to 151 U and then 368 U later in 2023.

Despite the reduction in CPK level, the patient experienced persistent muscle weakness and pain as well as exertional dyspnea and dysphagia, raising suspicion for fibro‐fatty muscle replacement. By late 2024, progressive orthopnea and reduced inspiratory capacity raised concern for progressively worsening diaphragmatic weakness.

### 2.2. Index Hospitalization and Treatment Course

The patient presented with respiratory distress in 2025 after receiving her last Gammagard infusion one week before hospitalization. Her manual muscle testing demonstrated neck flexion of 4/5, shoulder abduction of 4/5, hip flexion of 3/5, and knee extension of 3+/5, with preservation of strength in other muscles and a CPK of 81 U/L. Her negative inspiratory force (NIF) was −40 cm H_2_O, and her arterial blood gas analysis was consistent with acute on chronic hypercarbia. Pulmonary emboli were ruled out, and she had small pleural effusions and basilar atelectasis. BiPAP was begun, and the patient was transferred to an affiliated tertiary care hospital where myasthenia gravis and infection were ruled out and emergent PLEX (3 sessions over 5 days) was begun for presumptive diaphragmatic weakness.

A lower extremity MRI again showed diffuse fatty replacement of musculature with concomitant mild diffuse intramuscular edema consistent with active myositis. The patient’s respiratory mechanics continued to deteriorate, with NIF declining to −10 cm H_2_O on hospital day (HD) 8, requiring endotracheal intubation and initiation of mechanical ventilation (MV) for worsening hypercarbic respiratory failure. She also had evidence of acute kidney injury (AKI) due to acute tubular necrosis (ATN) that was associated with a drop in the GFR to a nadir of 16 mL/min/1.73 m^2^. The HMGCR antibody level was elevated to 201 U, and PLEX was continued through HD 10–12; intravenous methylprednisolone 100 mg daily was started on HD 9. Despite these measures, the patient remained ventilator‐dependent with no improvement in respiratory muscle weakness.

Due to the past history of refractory disease and concurrent AKI that precluded further use of IVIg, a renally adjusted tofacitinib dose (5 mg daily) was begun as salvage therapy on HD 12–13 and HD 16–35 along with daily methylprednisolone 100 mg on HD 9–20. Over the subsequent week, her weakness improved despite an upper gastrointestinal bleed necessitating transfusion. The patient demonstrated gradual recovery of both inspiratory effort and limb strength, and by HD 19, her muscle strength improved, with 4+/5 neck flexors, 3+/5 hip flexors, and 4/5 knee extensors. She was extubated and transitioned to BiPAP on the same day and was off supplemental oxygen by HD 25 with a NIF measurement of −40 cm H_2_O. Her glucocorticoids were progressively tapered, and by HD 40, she was on 40 mg prednisone daily. She was transitioned to inpatient rehabilitation on HD 28. Her treatment course and therapies are summarized in Figure 2. The patient tolerated tofacitinib therapy with no adverse events. She was discharged home with a final NIF measurement of −60 cm H_2_O.

**FIGURE 2:**
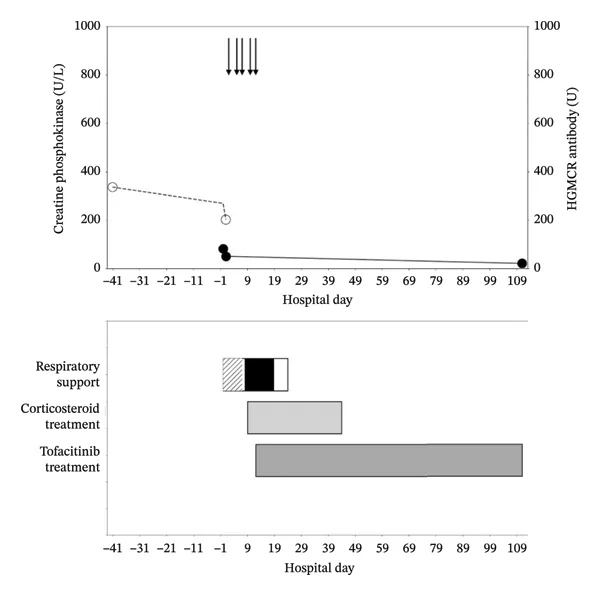
Index hospitalization and treatment course. (Top) CPK data points are denoted with black circles and a black solid line and HMGCR data points as open circles and a dashed line. Hospital day 0 is defined as the date of presentation to the emergency room, leading to index patient hospitalization. Solid black arrows denote days of performing plasma exchange (PLEX). (Bottom) Respiratory support: the dashed bar denotes biphasic positive airway pressure; the black bar denotes mechanical ventilation; and the white bar denotes supplemental oxygen by nasal cannula. Glucocorticoid treatment includes intravenous methylprednisolone and oral prednisone. Tofacitinib treatment was adjusted for renal function. Tofacitinib dosing was briefly interrupted for two days (HD 14–15) during the depicted treatment period; this brief interruption is not individually resolvable at the scale shown.

## 3. Discussion

This case illustrates the complexity of refractory HMGCR IMNM while highlighting the potential role of JAK inhibition in a patient who failed conventional immunosuppressive therapy, B cell depletion, and biweekly IVIg. Even with the use of PLEX, this patient demonstrated persistently elevated HMGCR antibody levels and profound muscle weakness.

A striking feature was the eventual discrepancy between CPK values and the degree of weakness at the time of her hospitalization with respiratory failure, likely related to the chronicity of her disease. Correspondingly, her MRI demonstrated concurrent features of muscle atrophy and damage along with muscle edema that was presumably indicative of some element of ongoing inflammation. Interestingly, the persistent elevation of HMGCR antibody titers likely correlated more closely with her clinical trajectory, underscoring the importance of not simply relying on the serum CPK level to monitor disease activity in advanced IMNM [9, 10].

The pivotal event in this case was the initiation of the JAK inhibitor tofacitinib, which temporally coincided with both respiratory recovery and increased muscle strength after failure of PLEX and high‐dose glucocorticoids. Within 1 week of starting tofacitinib, clinical improvement was noted, leading to a reversal of her rapidly declining respiratory muscle strength and allowing successful discontinuation of MV. The rationale for employing JAK inhibition in this context was derived from recent basic and translational science studies suggesting the existence of HMGCR‐antigen‐reactive CD4^+^ T cells in the circulation and affected muscles of patients, with an enrichment in T_H1_ and T_H17_ populations [11]. Additionally, in vitro studies previously demonstrated that coculture of muscle fibers with anti‐HMGCR autoantibodies was associated with production of TNF and IL‐6 and downregulation of IL‐4 and IL‐13, even in the absence of complement. Although bulk RNA sequencing identified low levels of Type 1 and 2 interferon (IFN1/IFN2) signaling in patient biopsies [12], infiltrating T cells and macrophages were skewed toward T_H1_ and M_1_ phenotypes, respectively, with upregulation of STAT1, IFNG, TNFA, and IL12 that suggested at least a modest role for IFN2 [13]. JAK inhibitors, such as tofacitinib, rapidly suppress downstream interferon signaling by blocking JAK1 and JAK3, thereby reducing transcription of interferon‐stimulated genes [14].

The clinical benefit of JAK inhibition has been reported in dermatomyositis, overlap myositis, and anti‐MDA5–positive disease, particularly in refractory cases with pulmonary or systemic involvement [8]—but has not been described in the setting of refractory IMNM. Our patient, who had exhausted multiple conventional therapies and PLEX, had no response to 4 days of steroid‐only treatment. Her rapid clinical improvement after initiation of a JAK inhibitor strongly suggests a therapeutic effect, although this cannot be definitively proven given that this was added to concurrent glucocorticoid therapy over the course of several weeks. Nevertheless, we believe that this case highlights the need for future study of JAK inhibitors in IMNM.

## Funding

No specific funding was received from any bodies in the public, commercial, or not‐for‐profit sectors to carry out the work described in this article.

## Ethics Statement

This case report has been thoroughly anonymized in accordance with the International Committee of Medical Journal Editors (ICMJE) recommendations, including generalization of the treating institutions, removal of month‐ and day‐level dates in favor of calendar year, and omission of nonessential identifying details. The authors’ institutional review board (IRB) was consulted regarding this matter and confirmed that single‐patient case reports of this nature do not constitute human subjects research and therefore fall outside its purview; accordingly, the IRB was unable to issue a determination in lieu of consent. All nonessential identifying patient details were omitted from this report to respect patient right to privacy. Written informed consent for publication was not obtained from the patient.

## Conflicts of Interest

L. Christopher‐Stine declares a conflict with Werfen and Inova Diagnostics for honoraria and intellectual property royalties related to HMGCR autoantibody testing. V. M. Liarski, A. Nicolas, and D. P. Ascherman are employees of VA Pittsburgh Healthcare System, Pittsburgh, PA. The contents do not represent the views of the U.S. Department of Veterans Affairs or the United States Government. The other authors declare no conflicts of interest.

## Data Availability

Data sharing is not applicable to this article as no datasets were generated or analyzed during the current study.
